# Religion and the Mediating Role of Alexithymia in the Mental Distress of Healthcare Workers During the Coronavirus Disease 2019 Pandemic in a Psychiatric Hospital in China

**DOI:** 10.3389/fpsyt.2022.837916

**Published:** 2022-04-25

**Authors:** Fushuai Zhao, Hsuan Lung, Po-Fei Chen, Mei-Chung Chang, For-Wey Lung

**Affiliations:** ^1^Anning Psychiatric Rehabilitation Hospital, Changchun Sixth Hospital, Changchun City, China; ^2^Department of Dentistry, Kaohsiung Chang Gung Memorial Hospital and Chang Gung University College of Medicine, Kaohsiung, Taiwan; ^3^Department of Psychology, Calo Psychiatric Center, Pingtung, Taiwan; ^4^Department of Nursing, Calo Psychiatric Center, Pingtung, Taiwan; ^5^Department of Medicine, Calo Psychiatric Center, Pingtung, Taiwan; ^6^Graduate Institute of Medical Sciences, National Defense Medical University, Taipei, Taiwan; ^7^International Graduate Program of Education and Human Development, National Sun Yat-sen University, Kaohsiung, Taiwan; ^8^Institute of Education, National Sun Yat-sen University, Kaohsiung, Taiwan

**Keywords:** coronavirus disease 2019, healthcare worker, mental distress, alexithymia, religion

## Abstract

The outbreak of the coronavirus disease 2019 (COVID-19) has created unprecedented challenges to the healthcare system, religion, and alexithymic trait that impacts the psychological resilience of healthcare workers during the COVID-19 pandemic. This study aimed to investigate the role religion and alexithymia play in mental distress and the level of happiness of psychiatric hospital healthcare workers in China amidst the COVID-19 pandemic. Furthermore, whether symptom dimensions (anxiety, depression, hostility, inferiority, and insomnia) are associated with the level of happiness, and a 6-month follow-up was also investigated. A total of one-hundred and ninety healthcare workers were recruited from a psychiatric hospital in Jilin, China, and 122 were followed up after 6 months. All participants filled out the 20-item Toronto Alexithymia Scale, five-item Brief-Symptom Rating Scale, and the Chinese Oxford Happiness Questionnaire. The mental distress of healthcare workers decreased from 2.6 to 1.5% in 6-months. Religious belief was not associated with the mental distress or happiness of healthcare workers. Instead, for those whose anxiety decreased over 6 months, their social adaptation status increased. For those whose inferiority level decreased over time, their perceived level of psychological well-being and overall happiness increased. In over half a century of living in different societies, religion stabilizes the mental health of those in Taiwan amidst the stress of the COVID-19 pandemic, but not in China. However, both regions found healthcare workers with alexithymic traits experienced a higher level of mental distress, implying that the collectivist culture of Confucian philosophy continues to influence the emotional expression and alexithymic traits of healthcare workers in China and Taiwan. To ensure a healthy and robust clinical workforce in the treatment and control of the pandemic, the cultural impact on the psychological resilience of medical workers needs to be addressed.

## Introduction

The outbreak of COVID-19 has created unprecedented challenges to the health care system globally. Increased stress experienced by healthcare workers has caused high levels of anxiety, depression, sleep disorders, burnout syndrome, and post-traumatic stress disorders (1), with a prevalence of up to 25.8–67.55% of anxiety, 24.3–55.89% of depression, and 45–62.99% of stress in systematic reviews (2, 3). Although compared to frontline healthcare workers who have direct care and contact with patients with COVID-19, healthcare workers working in psychiatric departments showed lower levels of mental distress (4). However, with the 4% high fatality rate of COVID-19 in China (5), there is an increased risk of mortality in patients with schizophrenia spectrum disorder (6). Medical staff in psychiatric hospitals also need extensive knowledge and relevant training in COVID-19 care (7).

Resilience is the ability of an individual to withstand setbacks, adapt positively, and recover from difficulties (8). Since healthcare professionals play an important role in the treatment and control of the pandemic, their mental and physical health conditions, and psychological resilience when faced with the pandemic becomes vitally important. Religion can help people develop coping strategies during stressful life situations (9), for it can provide social support, a healthy lifestyle, and meaning in life (10), and also plays a protective factor for mental health amidst the pandemic lockdown (11). Similarly, a previous study in Taiwan found religion to impact the mental health and level of happiness of healthcare workers, playing a vital role in the psychological resilience amidst the COVID-19 pandemic (4). Different emotional reactions and symptoms may appear at different periods of the pandemic. A previous study found anger post-disaster can predict psychological distress at follow-up, and hostility is high immediately post-disaster, but dissipate in a year (12). The five-item Brief Symptom Rating Scale (BSRS-5) measures the five symptoms of anxiety, depression, hostility, inferiority, and insomnia, and has been used to assess the mental distress of healthcare professionals in psychiatric and general hospitals (4). Therefore, the individual items within the BSRS-5 can also reflect different reactions under stress and stress reactions at different stages of the pandemic.

Besides different symptoms reactions, the alexithymic trait has also been shown to play a mediating role between COVID-19 exposure, posttraumatic stress disorder, and depressive symptoms (13). People who have alexithymic traits include those who have difficulty in identifying their feelings, differentiating feelings, verbalizing feelings, and communicating feelings (14). Alexithymia modulates the cortisol level in response to stressful events (15) and can predict the development of psychopathology during the pandemic (16). General and psychiatric hospital healthcare workers showed similar alexithymia levels, however, those healthcare workers that had alexithymic traits were more likely to experience mental distress and lower level of happiness (4). Therefore, the alexithymic trait is also an important predictor of psychological resilience during the COVID-19 pandemic among healthcare workers.

Since China and Taiwan share common cultural roots, traditions, and ancestries, but have lived in different societies for over half a century. A study in Taiwan showed religion and alexithymic trait both impacts the psychological resilience of healthcare workers during the COVID-19 pandemic (4). Therefore, the aim of this study was to investigate the role religion and alexithymia play in mental distress and the level of happiness of psychiatric hospital healthcare workers in China amidst the COVID-19 pandemic. Furthermore, whether symptom dimensions (anxiety, depression, hostility, inferiority, and insomnia) are associated with the level of happiness, and at 6-month follow-up was also investigated.

## Materials and Methods

### Participants

Healthcare workers, including administrative personnel, nurses, physicians, pharmacists, social workers, psychologists, radiologists, etc. from a psychiatric hospital in Jilin, China were conveniently recruited. The baseline questionnaires were collected from May 8th to June 1st, 2020 and followed up 6 months later (January 15th to February 1st of 2021). A total of one-hundred and ninety healthcare workers were recruited at the first stage and 122 (64.21%) at follow-up. Those who were unable to participate at the follow-up stage were due to the shifts of healthcare workers. The hospital has a total of 224 employees; thus our study had a response rate of 84.8%. The procedures performed in this study were approved by the Institutional Review Board of a teaching hospital in Taiwan, and informed consent was obtained from all participants after a detailed explanation of the study.

### Measurement

All information collected was from participants' self-report. The participants filled out the 20-item Toronto Alexithymia Scale (TAS-20), five-item Brief-Symptom Rating Scale (BSRS-5), and the Chinese Oxford Happiness Questionnaire at baseline (time = 1) and 6-month follow-up (time = 2). All the surveys collected were in Chinese and were of participants' self-report.

#### Religion

The major religion in China includes Buddhism, Taoism, Protestantism, Islam, Catholicism, and folk religions (17). Therefore, the demographic information sheet included the religious faith choices of “Buddhism/Taoism,” “Christian (Protestant)/Catholic,” “Shamanism” (local folk religion), and “others.”

#### Alexithymia

The Chinese version of the TAS-20 was translated from the original TAS-20 scale, developed to measure alexithymia in three dimensions: difficulty identifying feelings (DIF), difficulty describing feelings, and externally oriented thinking (18). Participants who scored ≧60 on the TAS-20 were considered to have alexithymia (19). Furthermore, those who score ≧21 in the DIF dimension have also been found to be at higher risk for psychiatric disorders (20). Therefore, the cutoff point of 60/61 for total TAS-20 and 21/22 for the DIF scale were both used in this study.

#### Mental Health Condition

The Chinese version of the BSRS-5 has been shown to be valid to screen for mental health conditions of psychiatric inpatients, general medical patients, and community residents in Taiwan (21). The BSRS-5 measures the mental health distress of participants in five symptom domains of anxiety, depression, hostility, interpersonal sensitivity/inferiority, and insomnia. The cutoff of 9/10 was valid to screen for healthcare workers who had higher psychological distress under the COVID-19 pandemic in Taiwan (4). Therefore, a cutoff of 9/10 was used in this study.

#### Happiness

The culturally modified seven-item Chinese Oxford Happiness Questionnaire was used to measure the self-perceived level of happiness of the healthcare workers. The culturally modified Chinese version of the happiness scale can be separated into two dimensions of social adaptation status (SAS; 4 items) and psychological well-being (PWB; three items) (22).

### Statistical Analysis

Descriptive analysis was used to analyze the demographic information and TAS-20, BSRS-5, and Chinese Oxford Happiness Questionnaire scores of the healthcare workers at the beginning of the pandemic and at the 6-months follow-up. Additionally, generalized equation estimation (GEE) analysis was used to analyze the factors which influenced the psychological resilience of the healthcare workers during the pandemic. GEE exchangeable covariance structure was chosen, it is the most suitable method of analysis for the measurement of repeated data. Parsimonious GEE models were presented, which means that only statistically significant (*p* ≤ 0.05) variables were presented. All analysis was processed using the Statistical Package for the Social Sciences (SPSS) 26.0 for Windows software (SPSS Inc., Chicago, USA).

## Results

The sociodemographic data, alexithymic traits, religion, psychological distress, perceived level of happiness of healthcare workers amidst the pandemic, and 6-month follow-up are shown in Table 1. Results showed a statistically significant difference in the total happiness scale between baseline and 6 months (*F* = 4.84, *p* = 0.29).

**Table 1:** Socio-demographic and clinical characteristics of healthcare workers at baseline and 6-months follow-up (*N* = 323).

	**Amidst** **the pandemic** ***n* = 191**	**6-months** **follow-up** ***n* = 132**	
**Variable**	***n*** **(%)**	***n*** **(%)**	**χ^2^**
Sex Male Female Department Medical Administrative Married Religious faith Buddhism/Taoism Christian/Catholic Shamanism Others No religion TAS-20 ≥61 TAS-DIF ≥22 BSRS-5 ≥10	39 (20.4) 152 (79.6) 162 (84.8) 29 (15.2) 88 (46.1) 10 (5.5) 4 (2.2) 2 (1.1) 47 (26.0) 118 (65.2) 5 (2.6) 13 (6.8) 5 (2.6)	29 (22.0) 103 (78.0) 112 (84.8) 20 (15.2) 67 (50.8) 6 (4.9) 3 (2.4) 1 (0.8) 33 (26.8) 80 (65.0) 5 (3.8) 3 (2.3) 2 (1.5)	0.11 <0.01 0.69 0.36 3.41 0.44
**Variable (range)**	**Mean (SD)**	**Mean (SD)**	***F***
Age (21–74) TAS-20 total score (20–80) BSRS-5 total score (0–20) Happiness Scale (11–28) Social adaptation status (5–16) Psychological well-being (3–12)	32.13 (10.0) 44.72 (8.8) 2.60 (2.9) 21.49 (2.8) 13.16 (1.7) 8.34 (1.8)	32.39 (9.0) 43.49 (8.9) 2.56 (2.9) 21.53 (3.4) 13.11 (2.0) 8.42 (2.0)	0.95 0.10 0.03 4.84* 1.81 2.34

TAS-20, 20-item Toronto Alexithymia Scale; TAS-DIF, Difficulty identifying feelings dimension of TAS-20; BSRS-5, five-item Brief-Symptom Rating Scale; *p < 0.05.

GEE was used to investigate which factors were associated with mental health distress level and perceived happiness, psychological well-being, and social adaptation status of these healthcare workers during the pandemic, and at 6 months follow-up. As Table 2 shows, religion was not associated with the mental health and perceived happiness of healthcare workers.

**Table 2:** Generalized equation estimation model of the association of religion on the mental health and level of happiness of healthcare workers over time.

**Dependent variable**	**Independent variable**	**ß**	**S.E**.	**95% C.I**.	***p***
BSRS	Religion	−0.06	0.45	−1.0 to 0.83	0.877
Perceived happiness	Religion	−0.30	0.54	−1.36 to 0.77	0.587
Psychological well-being	Religion	−0.09	0.37	−0.81 to 0.64	0.818
Social adaptation status	Religion	−0.22	0.25	−0.70 to 0.27	0.379

*BSRS-5, Five-item Brief-Symptom Rating Scale*.

The second GEE model investigated which factor was associated with the perceived happiness of the healthcare workers. Factors of interest included sex, age, religion, and mental distress level (BSRS total store). Since BSRS was the only factor associated with the perceived level of happiness of the healthcare workers, symptom domains of the BSRS (anxiety, depression, hostility, interpersonal sensitivity/inferiority, and insomnia) and its association with perceived happiness (including psychological well-being and social adaptation status) was further investigated in the second model. Additionally, symptoms domains that were shown to be associated with the perceived level of happiness, their interaction with time were also analyzed. The parsimonious results in Table 3 show the perceived level of happiness increased after 6 months (β = 0.68, *p* = 0.034). Of the five dimensions of the BSRS-5, those who had higher hostility levels perceived lower levels of happiness (β = −0.64, *p* = 0.002), and the interaction of inferiority and time showed higher inferiority levels over time also decreased the level of perceived happiness (β = −1.28, *p* = 0.001).

**Table 3:** Parsimonious generalized equation estimation model of the factors associated with the level of happiness of healthcare workers over time.

**Dependent variable**	**Independent variable**	**ß**	**S.E**.	**95% C.I**.	***p***
Perceived happiness	Time	0.68	0.32	0.05 to 1.31	0.034
	BSRS- Hostility	−0.64	0.21	−1.05 to −0.24	0.002
	BSRS-Inferiority	0.93	0.69	−0.43 to 2.29	0.180
	BSRS-Inferiority * Time	−1.28	0.39	−2.04 to −0.51	0.001
Psychological well-being	Time	0.37	0.19	<0.01 to 0.75	0.049
	BSRS-Inferiority	0.18	0.32	−0.46 to 0.81	0.586
	BSRS-Inferiority * Time	−0.50	0.19	−0.87 to −0.12	0.009
Social adaptation status	Time	0.21	0.21	−0.19 to 0.62	0.307
	BSRS-Anxiety	0.46	0.40	−0.33 to 1.25	0.255
	BSRS-Anxiety * Time	−0.66	0.27	−1.19 to −0.14	0.013
	BSRS-Inferiority	−0.40	0.15	−0.70 to −0.11	0.007

BSRS-5, Five-item Brief-Symptom Rating Scale; ^*^ interaction.

Regarding the psychological well-being dimension of the happiness scale, GEE results showed healthcare workers perceived better psychological well-being after 6 months (β = 0.37, *p* = 0.049). However, those who had higher inferiority level over time perceived lower levels of psychological well-being (β = −0.50, *p* = 0.009). On the other hand, no statistically significant differences between the baseline and follow-up level of social adaptation status were reported in healthcare workers. However, those who perceived a higher level of anxiety over time, and those who perceived a higher level of inferiority perceived a lower level of happiness at follow-up (β = −0.66, *p* = 0.013; β = −0.40, *p* = 0.007).

Finally, the third GEE model investigated the factors associated with mental distress level of healthcare workers. Factors of interest included sex, age, religion, and alexithymic trait (TAS-20 ≥61), and their interaction with time. The GEE model showed alexithymic trait was the only factor associated with the mental health distress level of the healthcare workers (Table 4). Those showing alexithymic traits (TAS ≥61) are at risk for higher levels of mental distress (β = −2.49, *p* = 0.003). Additionally, those who scored ≧22 in the DIF dimension of TAS-20 also experienced greater mental distress (β = −4.37, *p* = 0.001).

**Table 4:** Parsimonious generalized equation estimation model of the factors associated with the mental health distress level of healthcare workers.

**Dependent variable**	**Independent variable**	**ß**	**S.E**.	**95% C.I**.	***p***
BSRS	Time	−0.10	0.23	−0.55 to 0.36	0.679
	TAS-20 61	−2.49	0.84	−4.13 to −0.85	0.003
BSRS	Time	0.11	0.23	−0.34 to 0.56	0.625
	DIF 22	−4.37	1.27	−6.87 to −1.88	0.001

*TAS-20, 20-item Toronto Alexithymia Scale; DIF, Difficulty identifying feelings dimension of TAS-20*.

## Discussion

Our study showed, of the 191 healthcare workers in a psychiatric hospital in China, 2.6% reported having mental distress amidst the COVID-19 pandemic, and 1.5% at 6-months follow-up. Religion was not associated with mental distress or happiness in this group of healthcare workers. Instead, among the five symptom domains of anxiety, depression, hostility, inferiority, and insomnia, psychiatric healthcare workers who experienced higher hostility amidst the pandemic, perceived a lower level of happiness. The 6-month follow-up showed that inferiority decreased over time, which increased the perceived level of happiness and psychological well-being. In the same line, those who reported lower inferiority levels, perceived better social adaptation status. Besides inferiority, healthcare workers whose anxiety level decreased over the 6-month period, their social adaptation status also increased. Finally, those with alexithymic traits and/or who scored higher than 21 in the DIF dimension, experienced a higher level of mental distress compared to healthcare workers who did not have the alexithymic trait.

The level of mental distress amongst psychiatric healthcare workers was 2.6% amidst the pandemic, and lower (1.5%) at 6-months follow-up. This prevalence of mental distress is similar to the 2.96% reported in healthcare workers in psychiatric hospitals in Taiwan (4). However, this is much lower than the prevalence of 25.8–67.55% reported by healthcare professionals in systematic reviews (2), and 19.6 and 34.7% of anxiety and depression in the general public during the pandemic in China (23). The sampling period of the above systematic review and general population studies were earlier on in the pandemic, with the addition of information and experience of combating the pandemic from different countries, the level of distress of healthcare workers may have changed. Additionally, the distress level of healthcare workers also changes according to their regional incidence rates (24).

Amongst the symptoms of anxiety, depression, hostility, inferiority, and insomnia. Healthcare workers who reported a higher level of hostility perceived a lower level of happiness. The relationship between the level of hostile attribution and happiness is correlational (25), unhappy people may be prone to interpret ambiguous situations in an unfavorable way, which leads to negative emotions (anger) (26), and a lack of optimistic attributions may also lead to the low perceived level of psychological well-being (27).

This study also found those whose level of inferiority decreased over the period of 6 months, perceived better happiness and psychological well-being. In addition, those who reported lower inferiority levels, perceived better social adaptation status. This is in line with a previous study that found individuals with increased inferiority levels are more likely to self-concealment, which decreased their level of perceived happiness (28). Additionally, university students who spend more time participating in enjoyable activities of positive psychology reported lower levels of inferiority (29), which is associated with a higher level of subjective well-being (30).

The last symptom dimension associated with the happiness level of healthcare workers was anxiety. With healthcare workers whose anxiety level decreased over 6 months, associated with increased social adaptation status. Healthcare providers can generate remarkable stress and emotional turmoil during the outbreak of a pandemic like COVID-19 (31). Concerns about being infected and the possibility of putting the health of their family and friends at risk may cause healthcare workers to feel isolated and distressed (32). In addition, frontline medical personnel reports feeling less socially adapted compared with second-line medical personnel (33). Fortunately, a follow-up study in Taiwan also showed that the social adaptation status of healthcare workers increased over time (4).

No association was found between religion and mental distress or happiness in this group of healthcare workers. This result differed from a previous study that found religion as a psychological resilience factor among healthcare workers in Taiwan amidst the COVID-19 pandemic (4). These differences show that although China and Taiwan are of the same ethnic group, with common cultural roots, traditions, and ancestries. However, through the one-hundred-year process of social modernization in China (34), it was until the late 1970s that China adopted its policy of reform to open up political discourse and academic community on the topic of religion (35), as shown by less than ten percent of healthcare workers which reported to have religious faith in our study. In over half a century of living in different societies, religion stabilizes the mental health of those in Taiwan amidst the stress of the COVID-19 pandemic (4), but not in China. However, a previous study in China found a disparity in age and urbanization in the effect of religion on health, with religion significantly improving the health of urban residents and those over the age of 60 (36). However, another study also showed no association between religious belief and the health of elderly people (37). Showing inconsistent results in the impact of religion on health in China.

Although religious beliefs showed different impacts on the happiness of healthcare workers in China and Taiwan, however, both regions found healthcare workers with the alexithymic trait (TAS-20 ≥61) experienced a higher level of mental distress. This study further found those who scored over 22 in the DIF dimension of TAS-20 also experienced greater mental distress. This shows a collectivist culture of Confucian philosophy, encouraging the restraint of emotion, avoidance of interpersonal conflicts, and suppression of individual rights to maintain harmony with others continues to influence the emotional expression and alexithymic trait of healthcare workers in China and Taiwan. This cultural influence is also shown in the slower emotional development of children in a birth cohort study in Taiwan (38). Barella and Graffigna proposed that since healthcare professionals often have to deal with unexpected emotions from both patients and themselves, an emotional expression of healthcare providers may be considered unprofessional and inconvenient, implicitly encouraging clinicians' alexithymic traits to detach themselves from emotions (39). However, this alexithymic trait can influence the well-being of the healthcare providers, and the quality of medical care (40).

A limitation of this study was that data for this study were collected from one psychiatric hospital in China, therefore the generalizability of this study to other populations may be restricted. Especially since the psychological distress of healthcare workers in the epicenter of the pandemic were higher than those further from the epicenter (41), and the distress level of healthcare workers also changes according to the incidence rates in their region (24).

The strength of this study is that the mental distress and alexithymia levels of healthcare workers were followed-up over 6 months amidst the COVID-19 pandemic. Our follow-up study showed religious belief did not have an association with the mental distress of healthcare workers in China. Instead, the mental distress of healthcare workers decreased over time, and for those healthcare workers whose anxiety decreased over 6 months, their social adaptation status increased. Additionally, for those whose inferiority level decreased over time, their perceived level of psychological well-being and overall happiness increased. Healthcare workers with alexithymic traits were associated with a higher level of mental distress. Implementing strategies to assist healthcare workers with alexithymic traits in identifying their emotions and regulating their emotions can prevent or mitigate their mental distress. During a healthcare crisis, such as the COVID-19 pandemic, sharing emotions, concerns, and worries can make all those involved in the crisis feel more responsible and aware of how much their behavior can contribute to effectively coping with the stressful consequences of the situation (42). The Confucian and collectivist cultural impact on emotional expression needs to be considered. To ensure a healthy and robust clinical workforce in the treatment and control of the pandemic, policymakers should address the mental health needs of medical workers by funding preventive and promoting psychological resources (43), including spiritual resources and values for coping with the pandemic (44).

## Data Availability Statement

The raw data supporting the conclusions of this article will be made available by the authors, without undue reservation.

## Ethics Statement

The studies involving human participants were reviewed and approved by Kaohsiung Armed Forces General Hospital. The patients/participants provided their written informed consent to participate in this study.

## Author Contributions

HL and F-WL conceptualized the study. FZ and M-CC overlooked the sampling and experimental procedures. P-FC and F-WL undertook the statistical analysis and interpreted the analysis. P-FC wrote the first draft of the manuscript. All authors contributed to and have approved the final manuscript.

## Conflict of Interest

The authors declare that the research was conducted in the absence of any commercial or financial relationships that could be construed as a potential conflict of interest.

## Publisher's Note

All claims expressed in this article are solely those of the authors and do not necessarily represent those of their affiliated organizations, or those of the publisher, the editors and the reviewers. Any product that may be evaluated in this article, or claim that may be made by its manufacturer, is not guaranteed or endorsed by the publisher.
